# Kallmann syndrome and deafness: an uncommon combination: A case report and a literature review

**Published:** 2016-08

**Authors:** Nader Salama

**Affiliations:** 1 *Department of Surgery (Section of Urology), Taibah Faculty of Medicine, Taibah University, Al-Madinah, Saudi Arabia.*; 2 *Department of Urology, Alexandria Faculty of Medicine, Alexandria, Egypt.*

**Keywords:** *Kallmann syndrome*, *Deafness*, *Hypogonadism*

## Abstract

**Background:**

Kallmann syndrome (Kal S) is an isolated form of hypogonadotrophic hypogonadism in combination with a defect in smell sensation. Depending on the genetic form of the disease, a number of non-reproductive, non-olfactory abnormalities may also be existent. In the present report, we describe a male with Kal S associated with hearing loss, and the successful treatment of his sexual and reproductive defects.

**Case:**

A 23-year-old Caucasian man presented with a lifelong lack of erection and ejaculation. The patient reported also anosmia combined with loss of hearing ability. A diagnostic work-up identified the presence of Kal S associated with sensorineural hearing loss. Administration of gonadotrophins regained the erection and a viable-sperm containing ejaculation.

**Conclusion:**

Lack of erection and ejaculation are important components of delayed puberty which could lead to diagnosis of Kal S. The existence of a hearing impairment in the reported patient makes the recommendation to screen the hearing ability in Kal S of utmost importance.

## Introduction

The prevalence of Kallmann syndrome (Kal S) has been estimated to be approximately 1 in 8000 males. In females, the prevalence dropped about five times that in males (1). The real prevalence of Kal S is still, however, unknown. Kal S is a genetically heterogeneous disease. Its inheritance may be X-linked, autosomal dominant or autosomal recessive. At the time being, six genes have been identified and associated with the syndrome (*KAL1, FGFR1, PROK2, PROKR2, FGF8*, and *CHD7*) (2). 

Defects in these genes, nevertheless, were not demonstrated in the majority of the patients but in only 30% (2). A combination of hypogonadotrophic hypogonadism and anosmia typically highlights the possible existence of Kal S (1-3). In addition, the syndrome can be associated with a diversity of non-olfactory, non-reproductive features like renal and dental agenesis, cleft lip and palate, mirror movement and deafness (2, 4). However, the relationship between Kal S and hearing loss was sporadically reported and did not receive much attention. 

In this case report study, we describe a combination of delayed puberty and hearing inability in a male patient with Kal S, and the successful treatment of the patient’s sexual and reproductive disorders.

## Case report

A 23-year-old Caucasian consulted the Andrology Clinic at the Alexandria Main University Hospital for his small sized penis and lifelong inability to get any erection or ejaculation. Communication with the patient was done through his older brother as the patient had a primary deafness and an inability to speak. The brother mentioned the incapability of the patient to get erection whether during masturbation or with the aid of pornographic material. No morning erections were noticed. The same was also true for the ejaculation which could not happen with masturbation or during sleep. The patient was single and nonsmoker. He was illiterate with no chance to go to the school for education. He had a simple job in an oil company to depend on himself financially. He had no history of alcohol or drug intake. His mother gave birth for him without troubles in pregnancy or delivery. His past history was unremarkable. The patient was the fourth among 6 children in the family. His older and younger siblings were healthy with normal development. At birth, his father and mother were 31 and 28 years old, respectively with no history of consanguinity. His family history did not identify any abnormality except that his mother had a history of controlled epilepsy. The brother mentioned that their mother noticed the inability of the reported patient to differentiate between good (perfume) and bad (rotten stuff) odors since his childhood. The family noticed the obvious delay to initiate speech and later the hearing impairment of their son. 

Physical examination showed a slimy young man. He had a body weight, length and body mass index of 51.5 kg, 174 cm and 17, respectively. He had no gynecomastia. His secondary sexual characters were deficient. His flabby penis had 3 cm length and 2.5 cm mid-circumference. The testes were firm in consistency and very small in volume. The right and left testes were 0.3 cc and 0.4 cc in volume as estimated by scrotal ultrasound (normal average volume= 16 cc). The scrotal contents were normally palpated on both sides. There were no other physical abnormalities. Ultrasound examination of the abdominal organs did not show any relevant data. Hormonal assay identified that total testosterone=0.16 ng/mL (normal 2.8-8), free testosterone = 0.59 pg/mL (normal 4.5-42), leutinizing hormone=0.3 mIU/mL (normal 1.8-11.9), follicle-stimulating hormone (FSH)=0.4 mIU/mL (normal 0.7-11.1), prolactin=6.2 ng/mL (normal 2.7-13.7). Karyotyping showed 46XY pattern. It is recommended that karyotyping is done in males with hypogonadism and small-sized testes to rule out the possible existence of Klinefelter syndrome (5).

**Figure 1:**
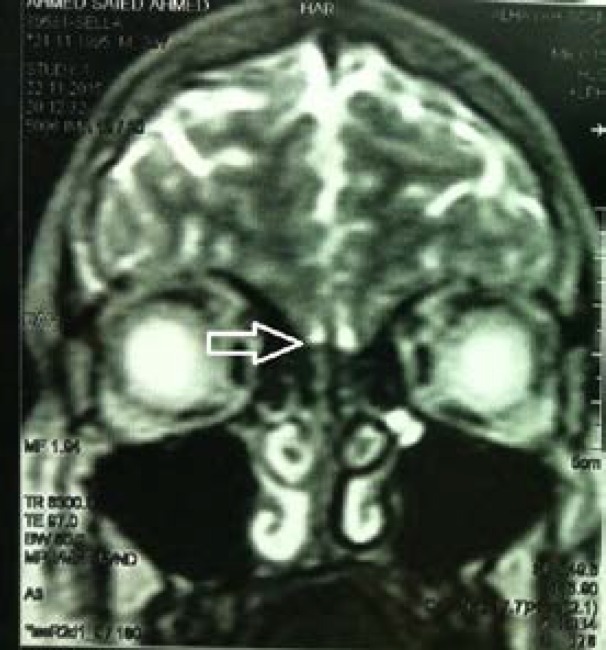
MRI scan of the brain identifying deficiency of the olfactory bulbs; the arrow denotes the presumed location of the olfactory bulbs

MRI scanning of the brain demonstrated absence of the olfactory bulbs (Figure 1). No other anomalies appeared on the scanning and the semicircular canals were normal. The patient was presumed to have Kal S (6, 7). Neuropsychological assessment was irrelevant and in particular, it did not disclose any mirror movements of the hands or the feet. The patient had also no intellectual disability. Evaluation of hearing inability of the patient was done using tympanometry showing sensorineural hearing impairment. An advice for a cochlear transplantation was given. 

The patient was kept on human chorionic gonadotrophin (hCG) 5000 IU once per week and FSH 75 IU twice per week. Six months later, there was an obvious development of the secondary sexual characters. The penis increased in length (5 cm) and girth (4.5 cm). Morning erections and wet dreams were reported. The patient was kept on the same treatment regimen for another 6 months. On the second follow-up visit, a semen analysis was done but it identified azoospermia. Nine months later using the same treatment protocol, the testes increased in volume (3 cc, each). 

A computer assessment sperm analysis (WHO, 2010) revealed semen volume: 2.5 cc, sperm count: 7 million/mL, sperm motility: 22% forward progression, 6% non-forward progression & 72% immotile populations and morphology index: 77.8%. A recommendation of sperm freezing was given. His new hormonal profile revealed that total testosterone=1.83 ng/mL, free testosterone= 15 pg/mL, LH=0.72 mIU/mL and FSH=0.85 mIU/mL. The patient was scheduled for regular follow-up regimen. 

## Discussion

Kal S is usually suggested clinically based on the combination of hypogonadism and anosmia/hyposmia (1-3). It is due to deficit in the olfactory bulb leading to incomplete migration of the neuroendocrine gonadotropin-releasing hormone (GnRH) cells into in the preoptic and hypothalamic regions (8). Diagnosis is usually delayed until the third decade when the patient seeks medical advice for delayed puberty and hypogonadism (1) as was the situation in the current case report. A number of non-olfactory, non-reproductive deficits, depending on the genetic form of the disease, have been described to frequently associate Kal S. These may comprise a big collection of disorders, including deafness (2, 4). 

Hearing impairment has been reported in a very limited number of males with Kal S (3, 7, 9). It was estimated by some workers to be 5% (1, 3). Hearing impairment in Kal S has been attributed to several possible genetic aberrations. These could include mutations in *KAL1*, *FGFR1*, *FGF8*, *IL17RD*, *CHD7 *and the transcriptional factor *SOX 10* which regulates development of neural crest cells, deletion in Xp22.3 where the gene responsible for X-linked Kal S resides or a single amino acid deletion of *KAL1* (3, 4, 7, 9, 10). The etiology in the majority of patients, however, is still unclear. Hearing inability in the currently-reported patient stopped his family to admit him to the school for education which subsequently had an implication on the quality of his life. We, therefore, agree with those pediatricians who recommended screening for hearing impairment in male children with micropenis or undescended testes, 2 features frequently associated with Kal S (3).

Hearing impairment in combination with hypogonadotrophic hypogonadism, anosmia and cleft palate are commonly seen in CHARGE syndrome which is caused by mutations in the *CHD7* gene (6). However, CHARGE syndrome can be excluded in our reported patient, although genetic screening was lacking. This exclusion is based on the lack of distortion of semicircular canals in our patient. These distortions are frequently apparent in CHARGE syndrome but unseen in Kal S (6, 7).

In the currently-reported patient, the main purpose was to treat the hypogonadism to induce the development of secondary sexual characters, and initiate spermatogenesis, ejaculation and erection. The hormone replacement therapy (FSH and hCG) given to the reported patient was successful in 2 aspects. 

First, biochemically to stimulate for testosterone production and raise the levels of the deficient gonadotropins. Second, clinically to treat the hypogonadism admitting verilization and spermatogenesis. This rendered the patient to develop erection and ejaculation in different occasions for the first time in his life. It also expedited the appearance of viable motile sperms in his ejaculate. Spontaneous pregnancies in couples, when the male partner has Kal S, have been reported (11). 

The current case study is the first reported case of Kal S associated with sensorineural deafness from the Middle East area. A limitation of this case presentation was the lack of genetic screening. However, mutation in any of the recognized 6 genes associated with Kal S was demonstrated in only 30% (2, 7) while mutation in* SOX10 was *displayed in 38% of the cases (4). This means that the inability to highlight any gene mutation does not preclude a diagnosis of Kal S. The diagnosis of the reported patient as a case of Kallmann syndrome was, therefore, purely clinical depending on the combination of anosmia and delayed puberty.

## Conclusion

We described a rare case of sensorineural hearing impairment in a male patient with Kal S. A verification of the common delay in diagnosis of the syndrome was also addressed. Boys with delay in initiating speech should be screened for hearing impairment and possibility to have Kal S, which may lead us to diagnose an individual genetic form of Kal S. Diagnosis of the Kal S at an early time, preferably before puberty, is specially advised to ensure a better quality of life. Treatment of hypogonadism associated with Kal S is definitely possible with good outcome for sexual and reproductive functions of the male patient. 
